# The prevalence and risk of developing major depression among individuals with subthreshold depression in the general population – ERRATUM

**DOI:** 10.1017/S0033291725100937

**Published:** 2025-07-07

**Authors:** Ruibin Zhang, Xiaoling Peng, Xiaoqi Song, Jixin Long, Chanyu Wang, Chichen Zhang, Ruiwang Huang, Tatia M. C. Lee

**Affiliations:** 1 Laboratory of Cognitive Control and Brain Healthy, Department of Psychology, School of Public Health, Southern Medical University, Guangzhou, China; 2 Department of Psychiatry, Zhujiang Hospital, Southern Medical University, Guangzhou, China; 3 Guangzhou Cana School, Guangzhou 510515, China; 4 School of Management, Southern Medical University, Guangzhou, China; 5 School of Psychology, South China Normal University, Guangzhou, China; 6 State Key Laboratory of Brain and Cognitive Sciences, The University of Hong Kong, Hong Kong, SAR China; 7 Laboratory of Neuropsychology and Human Neuroscience, The University of Hong Kong, Hong Kong, SAR China; 8 Center for Brain Science and Brain-Inspired Intelligence, Guangdong-Hong Kong-Macao Greater Bay Area, Guangzhou, China

When this article was originally published in Psychological Medicine it included the incorrect supplementary material. The correct supplementary material is now available online.

The publisher apologises for this error.
